# Supplementary material for: Exploring Cold Hardiness within a Butterfly Clade: Supercooling Ability and Polyol Profiles in European Satyrinae
Source: Insects. 2022 Apr 9;13(4):369. doi: 10.3390/insects13040369 (PMC9031891; doi:10.3390/insects13040369)

Supplementary material for

Exploring cold hardiness within a butterfly clade: supercooling ability and polyol profiles in European Satyrinae

By **Pavel Vrba, Alena Sucháčková Bartoňová, Miloš Andres, Oldřich Nedvěd, Petr Šimek** and **Martin Konvička**

Supplementary Table S1. Measured supercooling points (SCP) of all Satyrinae larvae assayed in this study, with information of survival or death after body fluids freezing.

Species	treatment	SCP (°C)	Survived the freezing
Coenonympha arcania	Autumn	-19.3	no
Coenonympha arcania	Autumn	-20.0	no
Coenonympha arcania	Autumn	-19.2	no
Coenonympha arcania	Autumn	-17.0	no
Coenonympha arcania	Autumn	-22.8	no
Coenonympha arcania	Autumn	-20.1	no
Coenonympha arcania	Autumn	-20.9	no
Coenonympha arcania	Autumn	-18.6	no
Coenonympha arcania	Autumn	-22.7	no
Coenonympha arcania	Autumn	-19.7	no
Coenonympha arcania	Autumn	-23.6	no
Coenonympha arcania	Autumn	-18.9	no
Coenonympha arcania	Autumn	-19.8	no
Coenonympha arcania	Autumn	-21.9	no
Coenonympha arcania	Autumn	-20.4	no
Coenonympha arcania	Autumn	-20.1	no
Coenonympha gardetta	Autumn	-20.9	no
Coenonympha gardetta	Autumn	-21.7	no
Coenonympha gardetta	Autumn	-19.8	no
Coenonympha gardetta	Autumn	-22.2	no
Coenonympha gardetta	Autumn	-21.9	no
Coenonympha gardetta	Autumn	-23.7	no
Coenonympha gardetta	Autumn	-22.1	no
Coenonympha gardetta	Autumn	-21.9	no
Coenonympha gardetta	Autumn	-23.0	no
Coenonympha gardetta	Autumn	-20.8	no
Coenonympha gardetta	Autumn	-19.3	no
Coenonympha gardetta	Autumn	-23.5	no
Coenonympha gardetta	Autumn	-21.4	no
Coenonympha gardetta	Autumn	-21.7	no
Coenonympha gardetta	Autumn	-21.9	no
Coenonympha gardetta	Autumn	-23.2	no
Maniola jurtina	Autumn	-15.1	no
Maniola jurtina	Autumn	-12.8	no
Maniola jurtina	Autumn	-15.0	no
Maniola jurtina	Autumn	-12.9	no

Maniola jurtina	Autumn	-7.2	yes
Maniola jurtina	Autumn	-6.4	yes
Maniola jurtina	Autumn	-7.0	yes
Maniola jurtina	Autumn	-13.3	no
Maniola jurtina	Autumn	-5.7	yes
Maniola jurtina	Autumn	-13.2	no
Maniola jurtina	Autumn	-13.4	no
Maniola jurtina	Autumn	-6.8	yes
Maniola jurtina	Autumn	-13.4	no
Maniola jurtina	Autumn	-13.0	no
Maniola jurtina	Autumn	-13.7	no
Maniola jurtina	Autumn	-12.6	no
Melanargia galathea	Autumn	-6.6	no
Melanargia galathea	Autumn	-7.3	no
Melanargia galathea	Autumn	-12.1	no
Melanargia galathea	Autumn	-13.7	no
Melanargia galathea	Autumn	-8.5	no
Melanargia galathea	Autumn	-7.4	no
Melanargia galathea	Autumn	-13.7	no
Melanargia galathea	Autumn	-14.6	no
Melanargia galathea	Autumn	-16.0	no
Melanargia galathea	Autumn	-12.7	no
Melanargia galathea	Autumn	-8.9	no
Melanargia galathea	Autumn	-13.1	no
Melanargia galathea	Autumn	-15.9	no
Melanargia galathea	Autumn	-7.6	no
Melanargia galathea	Autumn	-7.3	no
Melanargia galathea	Autumn	-6.9	no
Aphantopus hyperantus	Autumn	-6.4	yes
Aphantopus hyperantus	Autumn	-7.5	yes
Aphantopus hyperantus	Autumn	-8.0	yes
Aphantopus hyperantus	Autumn	-12.7	yes
Aphantopus hyperantus	Autumn	-10.3	yes
Aphantopus hyperantus	Autumn	-9.7	yes
Aphantopus hyperantus	Autumn	-6.5	yes
Aphantopus hyperantus	Autumn	-7.1	yes
Aphantopus hyperantus	Autumn	-12.7	yes
Aphantopus hyperantus	Autumn	-6.4	yes
Aphantopus hyperantus	Autumn	-7.5	yes
Aphantopus hyperantus	Autumn	-8.1	yes
Aphantopus hyperantus	Autumn	-7.2	yes
Aphantopus hyperantus	Autumn	-14.4	yes
Aphantopus hyperantus	Autumn	-13.4	yes
Aphantopus hyperantus	Autumn	-5.0	yes
Chazara briseis	Autumn	-21.7	no
Chazara briseis	Autumn	-20.3	no
Chazara briseis	Autumn	-18.0	no
Chazara briseis	Autumn	-25.4	no

Chazara briseis	Autumn	-22.2	no
Chazara briseis	Autumn	-19.0	no
Chazara briseis	Autumn	-21.1	no
Chazara briseis	Autumn	-25.0	no
Chazara briseis	Autumn	-22.8	no
Chazara briseis	Autumn	-19.8	no
Chazara briseis	Autumn	-23.3	no
Chazara briseis	Autumn	-20.7	no
Chazara briseis	Autumn	-25.0	no
Chazara briseis	Autumn	-23.4	no
Chazara briseis	Autumn	-18.9	no
Hipparchia semele	Autumn	-8.6	yes
Hipparchia semele	Autumn	-7.9	yes
Hipparchia semele	Autumn	-8.9	yes
Hipparchia semele	Autumn	-13.9	yes
Hipparchia semele	Autumn	-13.9	yes
Hipparchia semele	Autumn	-8.0	yes
Hipparchia semele	Autumn	-7.3	yes
Hipparchia semele	Autumn	-7.0	yes
Hipparchia semele	Autumn	-12.7	yes
Hipparchia semele	Autumn	-6.9	yes
Hipparchia semele	Autumn	-10.0	yes
Hipparchia semele	Autumn	-12.4	yes
Hipparchia semele	Autumn	-9.6	yes
Hipparchia semele	Autumn	-7.0	yes
Hipparchia semele	Autumn	-11.8	yes
Minois dryas	Autumn	-24.6	no
Minois dryas	Autumn	-20.4	no
Minois dryas	Autumn	-26.0	no
Minois dryas	Autumn	-22.7	no
Minois dryas	Autumn	-7.7	yes
Minois dryas	Autumn	-22.5	no
Minois dryas	Autumn	-18.7	no
Minois dryas	Autumn	-27.0	no
Minois dryas	Autumn	-25.3	no
Minois dryas	Autumn	-23.3	no
Minois dryas	Autumn	-7.1	yes
Minois dryas	Autumn	-22.2	no
Minois dryas	Autumn	-23.6	no
Minois dryas	Autumn	-21.3	no
Minois dryas	Autumn	-21.7	no
Coenonympha arcania	Winter	-21.1	no
Coenonympha arcania	Winter	-19.2	no
Coenonympha arcania	Winter	-19.1	no
Coenonympha arcania	Winter	-17.4	no
Coenonympha arcania	Winter	-22.5	no
Coenonympha arcania	Winter	-22.2	no
Coenonympha arcania	Winter	-18.8	no

Coenonympha arcania	Winter	-20.1	no
Coenonympha arcania	Winter	-21.9	no
Coenonympha arcania	Winter	-19.7	no
Coenonympha arcania	Winter	-20.6	no
Coenonympha arcania	Winter	-21.7	no
Coenonympha arcania	Winter	-22.6	no
Coenonympha arcania	Winter	-19.4	no
Coenonympha gardetta	Winter	-22.1	no
Coenonympha gardetta	Winter	-23.1	no
Coenonympha gardetta	Winter	-21.2	no
Coenonympha gardetta	Winter	-25.4	no
Coenonympha gardetta	Winter	-17.0	no
Coenonympha gardetta	Winter	-23.4	no
Coenonympha gardetta	Winter	-22.9	no
Coenonympha gardetta	Winter	-15.1	no
Coenonympha gardetta	Winter	-26.6	no
Coenonympha gardetta	Winter	-22.8	no
Coenonympha gardetta	Winter	-24.9	no
Coenonympha gardetta	Winter	-23.5	no
Coenonympha gardetta	Winter	-19.5	no
Coenonympha gardetta	Winter	-25.5	no
Coenonympha gardetta	Winter	-19.0	no
Coenonympha gardetta	Winter	-25.7	no
Melanargia galathea	Winter	-9.0	no
Melanargia galathea	Winter	-8.0	no
Melanargia galathea	Winter	-9.6	no
Melanargia galathea	Winter	-14.9	no
Melanargia galathea	Winter	-13.1	no
Melanargia galathea	Winter	-7.4	no
Melanargia galathea	Winter	-7.6	no
Melanargia galathea	Winter	-15.5	no
Melanargia galathea	Winter	-9.1	no
Melanargia galathea	Winter	-17.6	no
Melanargia galathea	Winter	-8.9	no
Melanargia galathea	Winter	-8.7	no
Melanargia galathea	Winter	-16.6	no
Melanargia galathea	Winter	-12.8	no
Melanargia galathea	Winter	-6.4	no
Melanargia galathea	Winter	-7.4	no
Aphantopus hyperantus	Winter	-8.9	yes
Aphantopus hyperantus	Winter	-7.1	yes
Aphantopus hyperantus	Winter	-9.7	yes
Aphantopus hyperantus	Winter	-13.9	yes
Aphantopus hyperantus	Winter	-7.5	yes
Aphantopus hyperantus	Winter	-8.2	yes
Aphantopus hyperantus	Winter	-7.3	yes
Aphantopus hyperantus	Winter	-15.2	yes
Aphantopus hyperantus	Winter	-8.0	yes

Aphantopus hyperantus	Winter	-8.6	yes
Aphantopus hyperantus	Winter	-14.2	yes
Aphantopus hyperantus	Winter	-8.5	yes
Aphantopus hyperantus	Winter	-8.8	yes
Aphantopus hyperantus	Winter	-9.7	yes
Aphantopus hyperantus	Winter	-9.7	yes
Aphantopus hyperantus	Winter	-8.8	yes
Maniola jurtina	Winter	-14.3	no
Maniola jurtina	Winter	-14.8	no
Maniola jurtina	Winter	-19.7	no
Maniola jurtina	Winter	-18.2	no
Maniola jurtina	Winter	-19.1	no
Maniola jurtina	Winter	-18.3	no
Maniola jurtina	Winter	-16.6	no
Maniola jurtina	Winter	-10.7	no
Maniola jurtina	Winter	-16.8	no
Maniola jurtina	Winter	-14.1	no
Maniola jurtina	Winter	-17.3	no
Maniola jurtina	Winter	-19.8	no
Maniola jurtina	Winter	-7.8	yes
Maniola jurtina	Winter	-15.0	no
Maniola jurtina	Winter	-11.1	no
Maniola jurtina	Winter	-20.5	no
Hipparchia semele	Winter	-9.2	yes
Hipparchia semele	Winter	-9.6	yes
Hipparchia semele	Winter	-8.3	yes
Hipparchia semele	Winter	-10.4	yes
Hipparchia semele	Winter	-9.2	yes
Hipparchia semele	Winter	-10.3	yes
Hipparchia semele	Winter	-10.5	yes
Hipparchia semele	Winter	-12.0	yes
Hipparchia semele	Winter	-7.8	yes
Hipparchia semele	Winter	-10.0	yes
Chazara briseis	Winter	-19.1	no
Chazara briseis	Winter	-25.7	no
Chazara briseis	Winter	-16.4	no
Chazara briseis	Winter	-10.9	no
Chazara briseis	Winter	-8.3	yes
Chazara briseis	Winter	-9.1	yes
Chazara briseis	Winter	-22.5	no
Chazara briseis	Winter	-21.1	no
Chazara briseis	Winter	-13.8	no
Chazara briseis	Winter	-24.8	no
Minois dryas	Winter	-23.1	no
Minois dryas	Winter	-23.5	no
Minois dryas	Winter	-19.5	no
Minois dryas	Winter	-12.3	no
Minois dryas	Winter	-18.8	no

Minois dryas	Winter	-26.5	no
Minois dryas	Winter	-21.4	no
Minois dryas	Winter	-18.5	no
Erebia medusa 2012	Autumn	-16.5	yes
Erebia medusa 2012	Autumn	-15	yes
Erebia medusa 2012	Autumn	-22	yes
Erebia medusa 2012	Autumn	-15	yes
Erebia medusa 2012	Autumn	-21	yes
Erebia medusa 2012	Autumn	-17	yes
Erebia medusa 2012	Autumn	-18	yes
Erebia medusa 2012	Autumn	-14	yes
Erebia medusa 2012	Autumn	-17	yes
Erebia medusa 2012	Autumn	-15.5	yes
Erebia medusa 2012	Autumn	-18	yes
Erebia medusa 2012	Autumn	-16.5	yes
Erebia medusa 2012	Autumn	-18.5	yes
Erebia medusa 2012	Autumn	-13	yes
Erebia medusa 2012	Autumn	-17	yes
Erebia medusa 2012	Autumn	-18	yes
Erebia epiphron	Autumn	-26.5	no
Erebia epiphron	Autumn	-20	no
Erebia epiphron	Autumn	-26.5	no
Erebia epiphron	Autumn	-18.5	no
Erebia epiphron	Autumn	-24.5	no
Erebia epiphron	Autumn	-15.5	no
Erebia epiphron	Autumn	-25.5	no
Erebia epiphron	Autumn	-21.5	no
Erebia epiphron	Autumn	-22.5	no
Erebia epiphron	Autumn	-25	no
Erebia epiphron	Autumn	-22.5	no
Erebia epiphron	Autumn	-19.5	no
Erebia epiphron	Autumn	-24.5	no
Erebia epiphron	Autumn	-20.5	no
Erebia epiphron	Autumn	-18.5	no
Erebia epiphron	Autumn	-21	no
Erebia sudetica	Autumn	-22	no
Erebia sudetica	Autumn	-7	no
Erebia sudetica	Autumn	-15	no
Erebia sudetica	Autumn	-9	no
Erebia sudetica	Autumn	-9.5	no
Erebia sudetica	Autumn	-10	no
Erebia sudetica	Autumn	-12.5	no
Erebia sudetica	Autumn	-15	no
Erebia sudetica	Autumn	-16.5	no
Erebia sudetica	Autumn	-17.5	no
Erebia sudetica	Autumn	-20	no
Erebia sudetica	Autumn	-15	no
Erebia sudetica	Autumn	-16	no

Erebia sudetica	Autumn	-17	no
Erebia sudetica	Autumn	-19.5	no
Erebia sudetica	Autumn	-20	no
Erebia tyndarus	Autumn	-5.5	no
Erebia tyndarus	Autumn	-8	no
Erebia tyndarus	Autumn	-5.5	no
Erebia tyndarus	Autumn	-9.5	no
Erebia tyndarus	Autumn	-8.5	no
Erebia tyndarus	Autumn	-11	no
Erebia tyndarus	Autumn	-7	no
Erebia tyndarus	Autumn	-11	no
Erebia tyndarus	Autumn	-6	no
Erebia tyndarus	Autumn	-15	no
Erebia tyndarus	Autumn	-8.5	no
Erebia tyndarus	Autumn	-6	no
Erebia tyndarus	Autumn	-5.5	no
Erebia tyndarus	Autumn	-12.5	no
Erebia tyndarus	Autumn	-8.5	no
Erebia tyndarus	Autumn	-6.5	no
Erebia medusa 2017	Autumn	-8.4	yes
Erebia medusa 2017	Autumn	-8.3	yes
Erebia medusa 2017	Autumn	-13.8	yes
Erebia medusa 2017	Autumn	-7.8	yes
Erebia medusa 2017	Autumn	-15.4	yes
Erebia medusa 2017	Autumn	-11.9	yes
Erebia medusa 2017	Autumn	-8.1	yes
Erebia medusa 2017	Autumn	-8.8	yes
Erebia medusa 2017	Autumn	-12.7	yes
Erebia medusa 2017	Autumn	-8.4	yes
Erebia medusa 2017	Autumn	-6.9	yes
Erebia medusa 2017	Autumn	-11.8	yes
Erebia medusa 2017	Autumn	-14.4	yes
Erebia medusa 2017	Autumn	-11.4	yes
Erebia medusa 2017	Autumn	-11.9	yes
Erebia medusa 2017	Autumn	-13.3	yes
Erebia medusa 2017	Winter	-8.4	yes
Erebia medusa 2017	Winter	-16.3	yes
Erebia medusa 2017	Winter	-19.1	yes
Erebia medusa 2017	Winter	-6.2	yes
Erebia medusa 2017	Winter	-8.2	yes
Erebia medusa 2017	Winter	-8.1	yes
Erebia medusa 2017	Winter	-8.8	yes
Erebia medusa 2017	Winter	-7.9	yes
Erebia medusa 2017	Winter	-18.0	yes
Erebia medusa 2017	Winter	-7.9	yes
Erebia medusa 2017	Winter	-11.5	yes
Erebia medusa 2017	Winter	-7.5	yes
Erebia medusa 2017	Winter	-21.8	yes

Erebia medusa 2017	Winter	-21.1	yes
Erebia medusa 2017	Winter	-7.3	yes
Erebia medusa 2017	Winter	-6.1	yes
Erebia aethiops	Autumn	-7.6	yes
Erebia aethiops	Autumn	-12.7	yes
Erebia aethiops	Autumn	-10.1	yes
Erebia aethiops	Autumn	-12.4	yes
Erebia aethiops	Autumn	-7.8	yes
Erebia aethiops	Autumn	-12.1	no
Erebia aethiops	Autumn	-23.3	no
Erebia aethiops	Autumn	-19.2	no
Erebia aethiops	Autumn	-16.2	no
Erebia aethiops	Autumn	-17.1	no
Erebia aethiops	Autumn	-12.3	no
Erebia aethiops	Autumn	-15.8	no
Erebia aethiops	Autumn	-18.8	no
Erebia aethiops	Autumn	-23	no
Erebia aethiops	Autumn	-22.6	no
Erebia aethiops	Autumn	-17.1	no
Erebia aethiops	Winter	-18.8	no
Erebia aethiops	Winter	-18.9	no
Erebia aethiops	Winter	-22.9	no
Erebia aethiops	Winter	-19.1	no
Erebia aethiops	Winter	-18.1	no
Erebia aethiops	Winter	-15.5	no
Erebia aethiops	Winter	-22.9	no
Erebia aethiops	Winter	-22.7	no
Erebia aethiops	Winter	-10.1	yes
Erebia aethiops	Winter	-12.6	yes
Erebia aethiops	Winter	-13.7	no
Erebia aethiops	Winter	-7.2	no
Erebia aethiops	Winter	-8.4	no
Erebia aethiops	Winter	-8.6	no
Erebia aethiops	Winter	-19.7	no
Erebia aethiops	Winter	-8.3	yes
Erebia pronoe	Autumn	-13.6	no
Erebia pronoe	Autumn	-23.5	no
Erebia pronoe	Autumn	-15.2	no
Erebia pronoe	Autumn	-12.1	no
Erebia pronoe	Autumn	-18.3	no
Erebia pronoe	Autumn	-19.0	no
Erebia pronoe	Autumn	-13.9	no
Erebia pronoe	Autumn	-13.9	no
Erebia pronoe	Autumn	-25.9	no
Erebia pronoe	Autumn	-18.3	no
Erebia pronoe	Autumn	-22.9	no
Erebia pronoe	Autumn	-20.4	no
Erebia pronoe	Autumn	-19.2	no

Erebia pronoe	Autumn	-24.8	no
Erebia pronoe	Autumn	-15.1	no
Erebia pronoe	Autumn	-23.8	no
Erebia pronoe	Winter	-9.5	no
Erebia pronoe	Winter	-24.4	no
Erebia pronoe	Winter	-16.8	no
Erebia pronoe	Winter	-12.0	no
Erebia pronoe	Winter	-4.8	no
Erebia pronoe	Winter	-8.2	no
Erebia tyndarus	Autumn	-25.3	no
Erebia tyndarus	Autumn	-24.6	no
Erebia tyndarus	Autumn	-23.1	no
Erebia tyndarus	Autumn	-23.3	no
Erebia tyndarus	Autumn	-24.1	no
Erebia tyndarus	Autumn	-24.2	no
Erebia tyndarus	Autumn	-26.1	no
Erebia tyndarus	Autumn	-15.3	no
Erebia tyndarus	Autumn	-23.7	no
Erebia tyndarus	Autumn	-18.4	no
Erebia tyndarus	Autumn	-23.4	no
Erebia tyndarus	Autumn	-22.2	no
Erebia tyndarus	Autumn	-19.5	no
Erebia tyndarus	Autumn	-17.2	no
Erebia tyndarus	Autumn	-23.1	no
Erebia tyndarus	Autumn	-14.1	no
Erebia tyndarus	Winter	-19.5	no
Erebia tyndarus	Winter	-13.8	no
Erebia tyndarus	Winter	-17.0	no
Erebia tyndarus	Winter	-18.5	no
Erebia tyndarus	Winter	-11.5	no
Erebia tyndarus	Winter	-17.2	no
Erebia tyndarus	Winter	-24.8	no
Erebia tyndarus	Winter	-15.9	no
Erebia tyndarus	Winter	-16.2	no
Erebia tyndarus	Winter	-19.9	no
Erebia tyndarus	Winter	-21.7	no
Erebia tyndarus	Winter	-19.4	no
Erebia tyndarus	Winter	-24.9	no
Erebia tyndarus	Winter	-25.3	no
Erebia pluto	Autumn	-7.4	no
Erebia pluto	Autumn	-15.8	no
Erebia pluto	Autumn	-17.5	no
Erebia pluto	Autumn	-21.6	no
Erebia pluto	Autumn	-8.2	no
Erebia pluto	Autumn	-9.1	no
Erebia pluto	Autumn	-8.6	no
Erebia pluto	Autumn	-18.2	no
Erebia pluto	Autumn	-14.6	no

Erebia pluto	Autumn	-17.0	no
Erebia pluto	Autumn	-24.9	no
Erebia pluto	Autumn	-10.1	no
Erebia pluto	Autumn	-16.3	no
Erebia pluto	Autumn	-16.7	no
Erebia pluto	Autumn	-25.9	no
Erebia pluto	Autumn	-24.3	no

Supplementary Table S2. Results of post-hoc Tukey's HSD tests used for multiple comparisons of SCP (**supercooling points, bold triangle**) and *TSPC (total sugars and polyols concentrations, triangle in italics)* measured on overwintering larvae of eight European monovoltine Satyrinae butterflies, obtained from ANOVA analysis with factors species and treatment (A and W: Autumn and Winter).

		C. arcania		***C. gardetta***		***M. jurtina***		***A. hyperantus***		***M. galathea***		***M. dryas***		***H. semele***		***C. briseis***	
		**A**	**W**	**A**	**W**	**A**	**W**	**A**	**W**	**A**	**W**	**A**	**W**	**A**	**W**	**A**	**W**
C. arcania	**A**		**1.00**	**0.99**	**0.94**	*	****	****	****	****	****	**0.99**	**0.60**	****	****	**1.00**	**1.00**
	**W**	*1.00*		**0.99**	**0.98**	*	****	****	****	****	****	**0.99**	**0.57**	****	****	**1.00**	**1.00**
C. gardetta	**A**	**	*0.11*		**1.00**	****	****	****	****	****	****	**1.00**	*	****	****	**1.00**	**0.99**
	**W**	*1.00*	*1.00*	*0.33*		****	****	****	****	****	****	**1.00**	*	****	****	**0.99**	**0.99**
M. jurtina	**A**	*0.95*	*0.99*	*0.67*	*1.00*		**1.00**	**1.00**	**1.00**	**0.80**	**0.99**	****	**	**0.99**	**0.99**	****	****
	**W**	*0.72*	*0.99*	*0.92*	*1.00*	*1.00*		**	**	****	****	**	**0.99**	****	***	**	**0.11**
A. hyperantus	**A**	****	***	*0.90*	***	**	*		**1.00**	**0.98**	**0.99**	****	****	**0.99**	**0.99**	****	****
	**W**	*0.10*	*0.55*	*1.00*	*0.86*	*0.99*	*0.99*	*0.40*		**0.97**	**0.99**	****	***	**0.99**	**1.00**	****	****
M. galathea	**A**	****	****	****	****	****	****	****	****		**1.00**	****	****	**0.99**	**1.00**	****	****
	**W**	*0.10*	*0.54*	*1.00*	*0.86*	*0.98*	*0.99*	*0.41*	*1.00*	****		****	****	**1.00**	**1.00**	****	****
M. dryas	**A**	****	****	****	****	****	****	****	****	*0.24*	****		**0.06**	****	****	**1.00**	**0.99**
	**W**	****	****	*0.17*	****	****	***	*0.97*	*	****	*	***		****	***	**0.29**	**0.79**
H. semele	**A**	*0.07*	*0.44*	*1.00*	*0.78*	*0.97*	*0.99*	*0.50*	*1.00*	****	*1.00*	****	*		**1.00**	****	****
	**W**	*0.0002*	**	*1.00*	*	*0.12*	*0.35*	*0.99*	*0.96*	****	*0.96*	****	*0.72*	*0.98*		****	****
C. briseis	**A**	***	*	*1.00*	*0.10*	*0.31*	*0.64*	*0.99*	*0.99*	****	*0.99*	****	*0.44*	*0.99*	*1.00*		**1.00**
	**W**	*0.99*	*1.00*	*0.27*	*1.00*	*1.00*	*0.99*	***	*0.80*	****	*0.80*	****	****	*0.71*	*	*0.07*	

Supplementary Table S3. Detailed results of correlations analyses (Pearson product moment correlation) relating concentrations of individual polyols in overwintering larvae (μg x g^{-1} fresh mass), plus their summed sugars and polyols concentrations (TSPC), to supercooling points (SCP) found for a selection of species of European univoltine Satyrinae butterflies. Concentration values were log-transformed to achieve normality of distributions.

Eight non-*Erebia* spp.

	**Both treatments (N = 16)**			**Autumn (N = 8)**			**Winter (N = 16)**		
	**r**	**t**	**P**	**r**	**t**	**P**	**r**	**t**	**P**
Glycerol	0.63	3.03	**	0.82	3.55	*	0.47	1.32	0.23
Ribose	-0.33	-1.30	0.21	-0.43	-1.17	0.29	-0.20	-0.49	0.64
Arabinitol	0.25	0.97	0.35	0.51	1.46	0.19	0.00	-0.01	1.00
Ribitol	0.00	0.00	1.00	-0.08	-0.19	0.86	0.12	0.29	0.78
Fructose	0.32	1.27	0.22	0.45	1.22	0.27	0.21	0.52	0.62
Glucose	-0.32	-1.25	0.23	-0.58	-1.76	0.13	0.15	0.38	0.71
Manitol	0.21	0.81	0.43	0.32	0.83	0.44	0.12	0.31	0.77
Sorbitol	-0.28	-1.10	0.29	-0.54	-1.58	0.16	0.18	0.44	0.67
Scyllo-inositol	0.06	0.23	0.82	-0.42	-1.12	0.31	0.47	1.32	0.24
Myo-inositol	0.45	1.87	+	0.56	1.68	0.14	0.26	0.65	0.54
Saccharose	0.53	2.33	*	0.60	1.82	0.12	0.48	1.34	0.23
Trehalose	-0.22	-0.82	0.42	-0.37	-0.98	0.36	0.10	0.25	0.81
Maltose	0.30	1.18	0.26	0.38	1.00	0.36	0.26	0.66	0.53
TSPC	0.20	0.77	0.46	0.14	0.34	0.75	0.36	0.95	0.38

Five *Erebia* spp. [15] added

	**Both treatments (N = 25)**			**Autumn (N = 13)**			**Winter (N = 12)**		
	**r**	**t**	**P**	**r**	**t**	**P**	**r**	**t**	**P**
Glycerol	0.02	0.10	0.92	-0.03	-0.10	0.92	0.15	0.48	0.64

Ribose	-0.22	-1.10	0.28	-0.25	-0.87	0.40	-0.25	-0.80	0.44
Arabinitol	0.15	0.71	0.49	0.04	0.13	0.90	0.25	0.83	0.43
Ribitol	0.05	0.23	0.82	-0.01	-0.03	0.98	0.11	0.37	0.72
Fructose	0.19	0.95	0.35	0.28	0.97	0.35	0.11	0.34	0.74
Glucose	-0.11	-0.54	0.59	-0.33	-1.17	0.27	0.23	0.75	0.47
Manitol	0.14	0.68	0.50	0.18	0.61	0.55	0.07	0.22	0.83
Sorbitol	-0.14	-0.66	0.52	-0.25	-0.87	0.40	-0.09	-0.30	0.77
Scyllo-inositol	0.14	0.67	0.51	-0.34	-1.20	0.26	0.40	1.36	0.20
Myo-inositol	0.40	2.12	*	0.52	2.03	+	0.20	0.65	0.53
Saccharose	0.45	2.44	*	0.56	2.27	*	0.38	1.30	0.22
Trehalose	-0.13	-0.62	0.54	-0.25	-0.84	0.42	0.00	-0.01	0.99
Maltose	0.23	1.14	0.27	0.21	0.71	0.49	0.31	1.04	0.32
Threitol	0.02	0.12	0.91	-0.03	-0.09	0.93	0.09	0.29	0.78
Erythritol	0.26	1.30	0.21	0.28	0.96	0.36	0.29	0.96	0.36
TSPC	0.06	0.31	0.76	0.02	0.08	0.94	0.13	0.40	0.70

Supplementary Table S4. Mean (±SD) concentrations (µg x mg^{-1} fresh mass) of sugars and polyols detected in the eight non-*Erebia spp.* targeted in this study, and six *Erebia spp*. targeted in [15]. Only substances reaching higher concentrations than 0.1 µg x mg^{-1} are listed. A and W: Autumn and Winter treatments.

		**Arabinitol**	**Fructose**	**Glucose**	**Glycerol**	**Maltose**	**Myo-Inositol**	**Ribitol**	**Saccharose**	**Sorbitol**	**Threitol**	**Trehalose**
C. arcania	A	**0.01**±0.00	**0.15**±0.04	**0.45**±0.13	**0.11**±0.03	**0.03**±0.02	**0.07**±0.05	**0.07**±0.05	**0.95**±0.82	**0.02**±0.00	**0**	**0.04**±0.01
	W	**0.01**±0.00	**0.13**±0.04	**0.47**±0.24	**0.09**±0.05	**0.01**±0.01	**0.07**±0.04	**0.04**±0.03	**1.55**±1.87	**0.01**±0.00	**0**	**0.07**±0.04
C. gardetta	A	**0.01**±0.00	**0.10**±0.04	**1.94**±0.93	**0.07**±0.02	**0.01**±0.01	**0.07**±0.04	**0.03**±0.02	**0.32**±0.55	**0.02**±0.01	**0**	**2.06**±0.36
	W	**0.02**±0.01	**0.23**±0.10	**1.06**±0.39	**0.05**±0.02	**0.00**±0.01	**0.09**±0.10	**0.01**±0.00	**0.01**±0.01	**0.03**±0.01	**0**	**1.26**±0.23
M. jurtina	A	**0.03**±0.01	**0.17**±0.04	**0.34**±0.08	**0.14**±0.02	**0.01**±0.01	**0.04**±0.03	**0.04**±0.04	**2.26**±0.55	**0**	**0**	**0.07**±0.02
	W	**0.04**±0.01	**0.61**±0.23	**1.10**±0.48	**0.17**±0.04	**0.03**±0.02	**0.07**±0.05	**0.03**±0.03	**1.25**±0.37	**0.03**±0.01	**0**	**0.07**±0.01
A. hyperantus	A	**0.02**±0.01	**0.18**±0.06	**0.57**±0.15	**0.16**±0.03	**0.03**±0.02	**0.37**±0.09	**0.01**±0.00	**3.22**±0.60	**0.02**±0.01	**0**	**1.34**±0.42
	W	**0.02**±0.01	**0.28**±0.09	**0.85**±0.21	**0.11**±0.02	**0**	**0.10**±0.09	**0.02**±0.01	**1.44**±0.33	**0.05**±0.02	**0**	**1.22**±0.33
M. galathea	A	**0.02**±0.01	**0.59**±0.09	**1.10**±0.32	**0.19**±0.05	**0.18**±0.02	**0.25**±0.12	**0**	**10.28**±2.75	**0**	**0**	**0**
	W	**0.02**±0.00	**0.78**±0.10	**1.87**±0.33	**0.15**±0.03	**0.15**±0.17	**0.19**±0.12	**0.01**±0.01	**0.68**±0.13	**0.09**±0.02	**0**	**0.15**±0.02
M. dryas	A	**0.01**±0.00	**0.54**±0.07	**2.35**±0.52	**0.06**±0.01	**0.02**±0.01	**0.07**±0.03	**0**	**6.23**±1.93	**0.07**±0.01	**0**	**1.31**±0.34
	W	**0.02**±0.00	**0.56**±0.23	**2.57**±0.87	**0.07**±0.02	**0.03**±0.05	**0.09**±0.03	**0**	**1.63**±1.16	**0.16**±0.06	**0**	**1.00**±0.53
H. semele	A	**0.01**±0.01	**0.29**±0.05	**1.08**±0.54	**0.11**±0.08	**0.01**±0.00	**0.07**±0.07	**0**	**2.27**±1.68	**0.03**±0.01	**0**	**0.33**±0.36
	W	**0.01**±0.00	**0.21**±0.17	**1.69**±1.59	**0.07**±0.04	**0.02**±0.02	**0.07**±0.04	**0**	**1.62**±0.94	**0.04**±0.01	**0**	**1.50**±1.25
C. briseis	A	**0.02**±0.01	**0.04**±0.01	**3.91**±1.22	**0.08**±0.03	**0.01**±0.00	**0.03**±0.03	**0**	**0.02**±0.06	**0.05**±0.01	**0**	**0.82**±0.59
	W	**0.01**±0.01	**0.04**±0.02	**1.42**±0.81	**0.06**±0.02	**0.01**±0.00	**0.02**±0.03	**0**	**0.19**±0.10	**0.07**±0.04	**0**	**0.87**±1.41
E. medusa	A	**0.04**±0.01	**0.04**±0.02	**1.29**±0.65	**0.27**±0.05	**0.02**±0.07	**0.03**±0.01	**0.11**±0.03	**1.58**±0.66	**0.00**±0.01	**0.14**±0.03	**4.07**±2.26
	W	**0.11**±0.04	**0.10**±0.09	**2.23**±1.31	**0.35**±0.09	**0**	**0.03**±0.01	**0.10**±0.05	**1.25**±0.77	**0.02**±0.02	**0.10**±0.04	**1.28**±1.35
E. aethiops	A	**0.03**±0.03	**0.35**±0.40	**0.60**±0.19	**0.40**±0.15	**0.02**±0.03	**0.03**±0.01	**0.04**±0.01	**1.25**±0.67	**0.03**±0.03	**0.11**±0.03	**0.88**±1.03
	W	**0.09**±0.07	**0.10**±0.05	**1.06**±0.44	**0.57**±0.22	**0**	**0.01**±0.01	**0.07**±0.03	**1.19**±0.49	**0.04**±0.04	**0.10**±0.03	**11.90**±3.12
E. pronoe	A	**0.02**±0.02	**0.08**±0.03	**0.42**±0.03	**0.68**±0.10	**0.13**±0.02	**0.02**±0.02	**0.01**±0.01	**0.69**±0.27	**0.01**±0.02	**0.07**±0.03	**0.95**±0.28
	W	**0.11**±0.03	**0.09**±0.03	**0.99**±0.34	**0.86**±0.05	**0**	**0.04**±0.01	**0.05**±0.01	**0.96**±0.27	**0.01**±0.02	**0.13**±0.02	**8.68**±0.88
E. cassioides	A	**0.04**±0.02	**0.02**±0.02	**0.43**±0.09	**0.51**±0.08	**0**	**0.02**±0.01	**0.06**±0.02	**0.45**±0.17	**0**	**0.12**±0.04	**11.28**±2.40
	W	**0.05**±0.02	**0.02**±0.02	**0.61**±0.24	**0.63**±0.06	**0**	**0.01**±0.01	**0.08**±0.02	**0.34**±0.24	**0**	**0.13**±0.05	**11.83**±3.64
E. pluto	A	**0.15**±0.12	**0.17**±0.06	**0.40**±0.20	**2.40**±2.75	**0.04**±0.05	**0.04**±0.01	**0.05**±0.01	**1.44**±0.20	**0.10**±0.03	**0.09**±0.07	**13.19**±1.90

Supplementary Figure S1. CCA ordination biplots (with first and second ordination axes) showing the sugars and polyols profiles in the eight non-*Erebia* (left) and satyrines including *Erebia* (right) if effect of treatment (Autumn vs. Winter) was filtered out as covariable (top), and if identities of species were treated as covariables (bottom). The letters H, I, and L denote High, Intermediate, or Low concentrations of individual polyols.

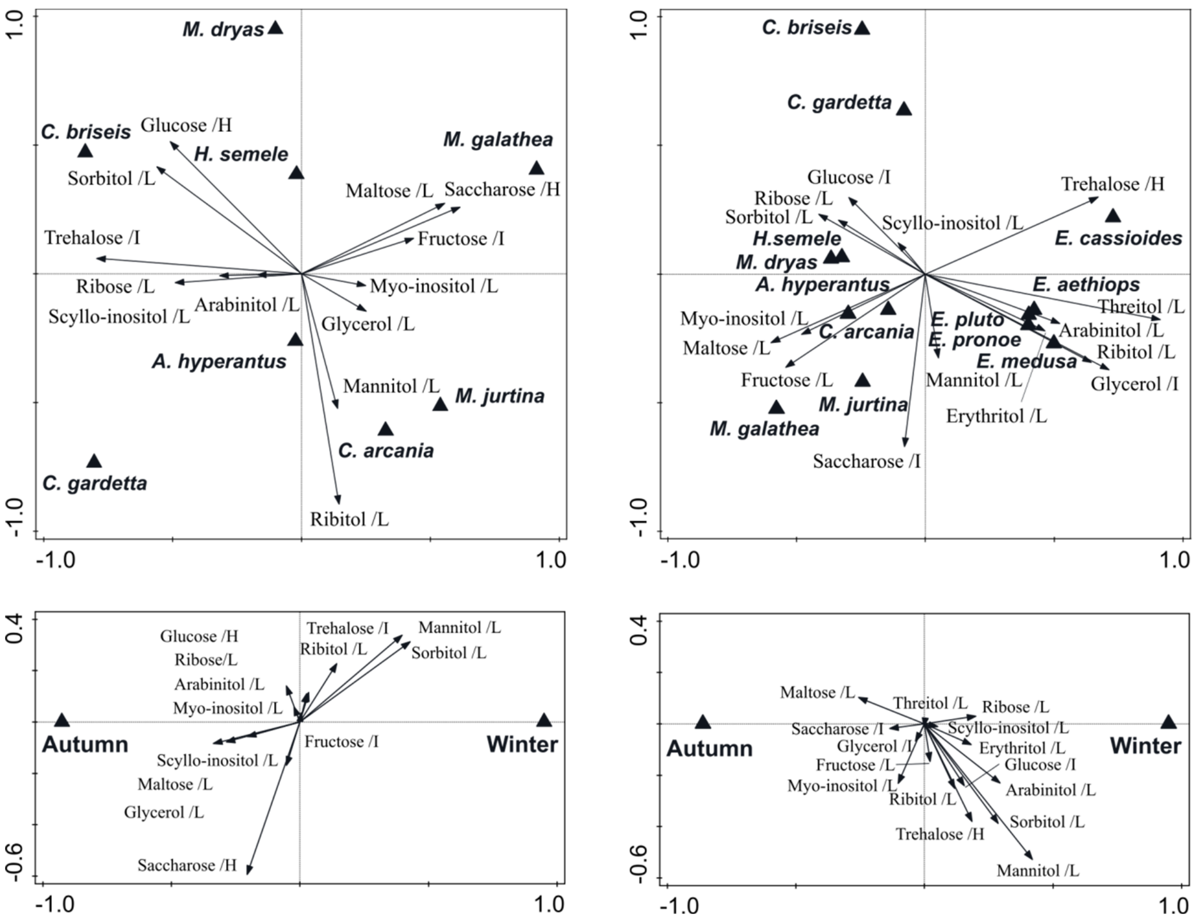

Supplement: Supplementary file 1 [file insects-13-00369-s001.zip › insects-1653504-supplementary.pdf]
